# ChatGPT as a Tool for Medical Education and Clinical Decision-Making on the Wards: Case Study

**DOI:** 10.2196/51346

**Published:** 2024-05-08

**Authors:** Anthony Skryd, Katharine Lawrence

**Affiliations:** 1 Department of Medicine NYU Langone Health New York City, NY United States; 2 Department of Population Health NYU Grossman School of Medicine New York City, NY United States

**Keywords:** ChatGPT, medical education, large language models, LLMs, clinical decision-making

## Abstract

**Background:**

Large language models (LLMs) are computational artificial intelligence systems with advanced natural language processing capabilities that have recently been popularized among health care students and educators due to their ability to provide real-time access to a vast amount of medical knowledge. The adoption of LLM technology into medical education and training has varied, and little empirical evidence exists to support its use in clinical teaching environments.

**Objective:**

The aim of the study is to identify and qualitatively evaluate potential use cases and limitations of LLM technology for real-time ward-based educational contexts.

**Methods:**

A brief, single-site exploratory evaluation of the publicly available ChatGPT-3.5 (OpenAI) was conducted by implementing the tool into the daily attending rounds of a general internal medicine inpatient service at a large urban academic medical center. ChatGPT was integrated into rounds via both structured and organic use, using the web-based “chatbot” style interface to interact with the LLM through conversational free-text and discrete queries. A qualitative approach using phenomenological inquiry was used to identify key insights related to the use of ChatGPT through analysis of ChatGPT conversation logs and associated shorthand notes from the clinical sessions.

**Results:**

Identified use cases for ChatGPT integration included addressing medical knowledge gaps through discrete medical knowledge inquiries, building differential diagnoses and engaging dual-process thinking, challenging medical axioms, using cognitive aids to support acute care decision-making, and improving complex care management by facilitating conversations with subspecialties. Potential additional uses included engaging in difficult conversations with patients, exploring ethical challenges and general medical ethics teaching, personal continuing medical education resources, developing ward-based teaching tools, supporting and automating clinical documentation, and supporting productivity and task management. LLM biases, misinformation, ethics, and health equity were identified as areas of concern and potential limitations to clinical and training use. A code of conduct on ethical and appropriate use was also developed to guide team usage on the wards.

**Conclusions:**

Overall, ChatGPT offers a novel tool to enhance ward-based learning through rapid information querying, second-order content exploration, and engaged team discussion regarding generated responses. More research is needed to fully understand contexts for educational use, particularly regarding the risks and limitations of the tool in clinical settings and its impacts on trainee development.

## Introduction

Large language models (LLMs) are computational artificial intelligence (AI) systems that are trained on large volumes of content from the internet and other sources to create natural, human-like written communications, images, and other outputs [1]. Popular general-use commercial LLMs include ChatGPT (OpenAI), Palm (Google), and LLaMA (Meta); health care–specific LLMs also in development but less widely available include Med-PaLM (Google), PMC-LLaMA (Meta), and BioGPT (Microsoft Corp) [2]. LLMs’ advanced natural language processing capabilities provide a unique opportunity to enhance medical education by providing students and educators with interactive, real-time access to and feedback on a vast amount of medical knowledge. This dialogue can be personalized to the level of the learner and leveraged to answer clinical questions, provide differential diagnoses, generate resources, and facilitate the assimilation of complex medical concepts [3]. Studies have documented facilities of ChatGPTs and others with standardized examinations, academic abstracts, and clinical documentation [4-6]. At the same time, general concerns have been raised regarding the practical usability, clinical practice implications, and overall ethics of using LLMs in health care [7,8]; for medical education, issues of overreliance, plagiarism, misinformation, bias, and inequity are particularly acute [9]. As a result, the adoption of LLM technology into medical education and training has been varied, and comprehensive guidelines for its systematic application in learning contexts remain underdeveloped.

In this case study, we present the use of a publicly available LLM (ChatGPT) as a real-time interactive educational tool for attending teaching rounds on an inpatient resident medicine service at a large urban academic medical center. We identify select ChatGPT use cases and qualitatively evaluate the tool’s impact on real-time clinical learning, diagnostic reasoning, and medical decision-making for medical residents and teaching attendings. We further explore the perceived advantages, limitations, and ethical considerations of ChatGPT in real-world clinical and teaching contexts and consider the future implications of generative AI conversational technology as a tool for medical education.

## Methods

### Context

We conducted a brief, single-site pilot study of the publicly available ChatGPT-3.5 (OpenAI) by implementing the tool in the attending rounds workflow of an inpatient general internal medicine service in a large urban academic medical center in New York.

Attending-style rounds were conducted for 1.5-2 hours daily in the mornings over the course of the 7-day rotation, consisting of patient presentations, case reviews, case-based learning, and didactics. ChatGPT was integrated into rounds via both structured and organic use, using the web-based “chatbot” style user interface to interact with the LLM through conversational free-text and discrete queries. ChatGPT was prompted via a zero-shot approach, without the use of prior training sets, data, or examples.

Before initiating the pilot study, the team established a code of conduct for ChatGPT’s use based on a shared understanding of its potential, general risks, and implications for patient care (Textbox 1). This code of conduct guided the tool’s use throughout the test period.

Over the 7 days of the pilot study, the team established a standardized implementation method and ensured adherence to the code of conduct for use. All relevant ChatGPT outputs were independently verified by team members using validated resources (eg, PubMed, UpToDate, and medical society guidelines). Any paper citations or references provided by ChatGPT were also reviewed.

**Team-developed code of conduct for ChatGPT use on attending rounds.**

We acknowledge the potential risks and harms of using technology like ChatGPT in our clinical work and training.

As a team, we agree:

- Not to use any specific or identifiable patient information in our interactions with ChatGPT.
- To independently verify and validate any answers ChatGPT produces, and not make medical decisions based on ChatGPT’s outputs unless we can confirm their accuracy.
- To be honest with our patients and one another when we use ChatGPT in our clinical work.
- To be open to the possibility that ChatGPT is more intelligent than we are.

Overall, we commit to placing patient care, safety, and trust above any educational or research use of this technology. We further commit to abiding by our institution’s information technology policies and practices.

### Analysis

A qualitative approach using phenomenological inquiry was used to identify key insights related to the use of ChatGPT for real-time ward-based educational contexts. Analysis was conducted in two phases: (1) an in situ review and validation of the ChatGPT outputs by the clinical team and (2) a retrospective rapid qualitative analysis using phenomenological inquiry performed by the coauthors (AS and KL) [10]. In phase 1, ChatGPT conversation logs and outputs were group-reviewed by the clinical team and qualitatively assessed for factualness, quality, relevance, and usefulness in clinical contexts. The team also conducted a group debrief at the culmination of the clinical block via a semistructured group interview led by the attending, which explored primary uses, perceptions, and learning from the experience and perceived impacts on medical education. In phase 2, the senior author (KL) used a pragmatic phenomenological approach to concisely review the ChatGPT conversation logs and associated short-hand attending notes from the clinical sessions and identify both emergent major, minor, and outlier themes and themes specifically related to the use of ChatGPT for real-time ward-based educational contexts; these themes were reviewed and revised between both authors (KL and AS) [10,11].

### Ethical Considerations

This study was approved as part of a quality improvement initiative under the NYU Grossman School of Medicine institutional review board. Study data do not include any personal health information related to any care provision conducted during the course of the investigation.

## Results

### Overview

The team was comprised of 7 members: 1 attending internal medicine physician, 1 senior medicine resident, 2 interns, 2 medical students, and 1 physician assistant student. All team members expressed baseline familiarity with ChatGPT, with most knowledge derived from social and general media information, particularly around controversies in its use. No team member had received formal ChatGPT training or guidance on its use from their educational program or was actively using the technology in their clinical work. No team member had prior experience with prompt engineering, specific coding, or data management skills to interact with the tool at a more advanced level.

Over the course of the 7-day pilot study, ChatGPT was queried 17 times, representing a combination of single-question queries and longer bidirectional interchanges. ChatGPT prompts were generated by all members of the team. The types of ChatGPT use during attending rounds were identified (Textbox 2).

**ChatGPT use cases and examples identified by the study team during use on the wards.**

**Discrete medical knowledge inquiries**

- Review of common illnesses
- Uncommon diagnoses
- Clinical aids and diagnostic calculators
- Medication interactions and side effects
- Mechanism of action of medications

**Building differential diagnoses and engaging dual-process thinking**

- Initial and expanded differential diagnoses
- Rare diagnoses
- Validating clinical reasoning

**Challenging medical axioms**

- Validating existing knowledge
- Challenging teaching points

**Cognitive aids in acute care scenarios**

- Initial evaluation
- Further testing considerations
- Other cognitive aids
- Debriefing tools

**Facilitating conversations with subspecialties**

- Identifying specialty-specific best practices to cite when discussing a complex case with multiple specialties involved
- Patient advocacy resources

**Other topics**

- Engaging in difficult conversations
- Ethical challenges and general medical ethics
- Personal continuing medical education resources
- Development of teaching tools
- Clinical documentation
- Productivity and task management support

### Discrete Medical Knowledge Inquiries

Attending rounds generated numerous discrete medical knowledge questions, often based on the knowledge gaps of team members or inquiries from specific patient cases. During this pilot, ChatGPT was substituted for other frequently used web-based resources (eg, UpToDate) to answer many discrete medical inquiries; this represented a combination of first-order (factual information seeking) and second-order (process and reasoning seeking) questions (Textbox 2 and Figures S1-S3 in Multimedia Appendix 1), with first-order questions frequently leading to additional second-order queries. In general, using ChatGPT for discrete knowledge inquiries often led to further questions and additional prompting, in turn generating team discussion and knowledge sharing. This process was identified by both the attending and the junior team members as an effective way to gain additional knowledge regarding a topic of interest without considerable extra time or effort.

### Building Differential Diagnoses and Engaging Dual-Process Thinking

Crafting a comprehensive differential diagnosis is essential to clinical reasoning in patient evaluation [12]. A well-known method to engage complex reasoning is the dual-process approach [13], whereby system 1 reflexive, intuitive thinking is complemented by system 2 rational and more cognitively intensive analytic thought [14,15]. During the pilot study, ChatGPT was regularly queried to provide differential diagnoses for general patient presentations, with additional prompting to provide expanded differentials, including uncommon diagnoses. The team reviewed these differentials and discussed their plausibility, likelihood in the case context, and completeness compared to the team-generated differentials. In most instances, the differentials provided by ChatGPT mirrored those of the medical team; in some cases, differentials collaboratively produced by team members were more expansive and included likely diagnoses not provided by ChatGPT (Figure S4 in Multimedia Appendix 1), while in fewer instances, ChatGPT provided novel diagnoses that prompted additional queries and resulted in new learning for the team (Figures S5 and S6 in Multimedia Appendix 1). Overall, the team reflected that ChatGPT-generated differentials were considered a helpful supplement to team-generated ones for confirming clinical reasoning and completeness but did not meaningfully change leading diagnoses or care plans.

### Challenging Medical Axioms

An axiom is defined as a rule, principle, or truth that is often widely accepted on its merit without proof or basis for further analysis [16]. In this case, ChatGPT was used to query axiomatic practice on rounds and either justify or challenge them (Figures S7-S9 in Multimedia Appendix 1). Common practice habits (eg, timing of medication doses) or practices used widely across patients (eg, electrolyte repletion) were queried most often—generally in response to a team member’s question (Why do we do this?)—resulting in deeper knowledge for the team regarding their daily work. The use of ChatGPT to confirm or challenge medical axioms generated a thoughtful discussion among the team members regarding the basis of medical knowledge and the transmission of learning in medical education.

### Cognitive Aids in Acute Care Scenarios

Acute care scenarios and medical emergencies are stressful, complex events that require rapid response and coordination of care that is often time-critical. The use of cognitive aids and checklists has been widely studied and proven effective in optimizing care and reducing errors in emergency response scenarios [17]. During the pilot study, ChatGPT was deployed as part of a rapid response team (RRT) debrief, in which the tool was used after the team had performed an RRT to replicate and review the clinical scenario (Figures S10 and S11 in Multimedia Appendix 1). On review, the overall structure of ChatGPT’s RRT management response (eg, initial scene assessment and triage process) was considered poorly organized and insufficiently specific to guide real-time RRT management. Conversely, outputs did include thorough reasoning for recommended RRT procedures (eg, laboratory testing and imaging), which the team perceived as helpful to recall during high-stress scenarios (Figure S11 in Multimedia Appendix 1). Overall, the team felt the level of prompting and interaction required to get appropriate and well-structured information to guide an RRT was overly burdensome and inefficient compared to existing processes.

### Facilitating Conversations With Subspecialties

Another use for ChatGPT was identified during a complex patient case involving multiple specialties and ongoing goals of care conversation. During rounds, the team expressed concern regarding their ability to effectively care for the patient and their communications with specialists as a result. In response, ChatGPT was queried regarding specific best practice guidelines for medical versus surgical management of the condition (Figures S12 and S13 in Multimedia Appendix 1). Outputs provided by ChatGPT were supplemented with web-based inquiry to validate the content and identify the most up-to-date information; through this combined internet and ChatGPT process, a previously unknown set of guidelines (Figure S14 in Multimedia Appendix 1) [18] were identified, which provided the team with specialty-specific references to guide further high-level conversations with consultants. Overall, the team felt that ChatGPT had given them a better understanding of management options, which empowered them to advocate for their patient and work collaboratively with specialists on the case.

### Other Topics

Upon completing the 7-day pilot study, the team met to debrief on the experience and identify additional potential use cases. These included engaging in difficult conversations with patients, exploring ethical challenges and general medical ethics teaching, personal continuing medical education resources, developing teaching tools (eg, “teaching on the wards” aids), supporting and automating clinical documentation, and supporting productivity and task management. Of note, when prompted throughout the investigation to cite specific references, ChatGPT provided outputs but noted it could not cite the specific location of the information provided; further review of the citations by the team revealed none referred to actual papers. Reference provisions were therefore considered unreliable use case by the team. The team also discussed ongoing ethical issues in ChatGPT uses for health care, including its well-documented biases and potential to result in health inequities, medico-legal implications, data privacy and security, and potential nefarious uses. This discussion resulted in a prompt inquiry to ChatGPT on the future of medical education as generative AI technologies advance (Figure S15 in Multimedia Appendix 1).

## Discussion

### Principal Findings

This exploratory case study examined various use cases of the commercially available LLM, ChatGPT, as an educational tool for attending teaching rounds of an inpatient resident medicine service at a large urban academic medical center. We identified several key areas for which ChatGPT was used, including addressing team or individual knowledge gaps and validating funds of knowledge through discrete medical knowledge inquiries; expanding differential diagnoses and engaging dual systems process thinking to validate and expand clinical reasoning; challenging axioms through active investigation of default medical knowledge and practice heuristics; supporting triage, diagnostic, and care decision-making during acute care emergencies; and facilitating patient advocacy and complex care management through improved specialty consultations. Other topics not directly explored but identified as potential use cases included challenging patient scenarios and conflicts, medical ethics inquiries, general continuing medical education, and team productivity and efficiency. Overall, the tool was considered a promising addition to the learning environment, while it was also noted to be limited in accuracy, reliability, and usability in its current state. In particular, using ChatGPT as a real-time educational aid during attending rounds enhanced team learning by fostering a discussion of responses and generating further areas of exploration and inquiry.

### Contributions to the Literature and Limitations

While exploratory, this case study adds to the rapidly growing literature exploring the various uses and limitations of generative AI technologies, such as LLMs, in medical education. As previously stated, ChatGPT has been successfully tested in a growing number of medical training contexts, including writing medical notes and academic abstracts and completing the United States Medical Licensing Examination (USMLE); a surfeit of opportunities to explore the impact of generative AI tools in medical education has also been identified at the undergraduate, graduate, and professional levels [5,6,19-22]. Significant work remains, however, to better understand the roles of these technologies in medical education, including both the extent to which these technologies have *already* been integrated into educational programs (either formally or through casual use) as well as the true appetite for their future use [23,24]. At the same time, there is an ongoing need to better characterize and actively mitigate the risks of these tools’ use in care delivery, particularly among developing trainees. This study identified numerous limitations of the technology, many of which have been described elsewhere. These included difficulties validating information sources and specific references, inconsistent responses to similar prompts, and incomplete access to major databases and up-to-date material [19]. Although we were unable to confirm examples of misinformation or bias—such as the generated missing references—that may have occurred during our pilot study, our experience reflects larger grievances with current publicly available LLM tools, in particular around issues of output “trustworthiness” and response fidelity over time [25]. There is also the potential of ChatGPT and other tools to perpetuate cognitive and sociocultural biases [26,27], impacting both trainee development and overall care delivery; processes to better center equity in LLMs and mitigate bias-related AI harms are needed to address this [1,28,29].

In addition to the larger issues of LLMs identified earlier, this study has several important limitations. Using ChatGPT on a single team over the 7 days of a shared clinical rotation restricted the depth and range of analysis to a small-scale pilot study with a short duration; future investigations should extend this period as well as evaluate the impact off different clinical care teams (eg, nurses and pharmacists) and other team factors. Significantly, our team had limited knowledge of prompt engineering and optimization in generating desirable ChatGPT outputs, which likely limited our interaction potential with technology and may have introduced specific interaction biases; conversely, our interactions with the ChatGPT user interface also likely represent an “average” user experience for a health care provider at the time, which will likely evolve as clinicians gain familiarity and skill with the tool. Future work should emphasize the role of prompt engineering and various other approaches to priming and interacting with LLMs (eg, “zero-shot” vs few-shot learning) [30]. Additionally, ChatGPT output quality was assessed without the aid of existing validated tools to measure performance or in comparison to other LLMs; there is a need for both general objective measures as well as comparative metrics across products to more rigorously benchmark and compare these tools.

### Conclusions

This case study explored ChatGPT as an educational tool in an inpatient academic medical service. Several noteworthy use cases of LLM were identified, including addressing knowledge gaps, expanding differential diagnoses, challenging medical axioms, supporting acute care decision-making, and facilitating complex care management through improved specialty consultations. Overall, ChatGPT enhanced team learning by prompting engaged discussion and further areas of exploration and inquiry. LLMs continue to demonstrate promise and peril in health care, with particular opportunities and risks in educational spaces. Concerns related to biases, misinformation, and ethical implications in health care emphasize the need for further consideration and regulatory guidelines for LLM application in medical education and clinical practice. Ultimately, technical progress (or stasis), regulatory oversight, and social appetite will likely decide their future. Researchers should continue to study impacts by identifying further use cases for investigation, conducting meta-analyses on the myriad of case studies currently being conducted, better defining study designs and evaluation tools, and advocating for the safe, ethical, and equitable use of these technologies.

## Appendix

Multimedia Appendix 1 Sample ChatGPT queries and outputs.

## Abbreviations

AI: artificial intelligence
LLM: large language model
RRT: rapid response team

## Data Availability

The data sets generated and analyzed during this study are available from the corresponding author on reasonable request.
