# Supplementary material for: ChatGPT as a Tool for Medical Education and Clinical Decision-Making on the Wards: Case Study
Source: JMIR Form Res. 2024 May 8;8:e51346. doi: 10.2196/51346 (PMC11112466; doi:10.2196/51346)
Supplement: Multimedia Appendix 1 [file formative_v8i1e51346_app1.docx]

Multimedia Appendix 1. Sample ChatGPT queries and outputs.


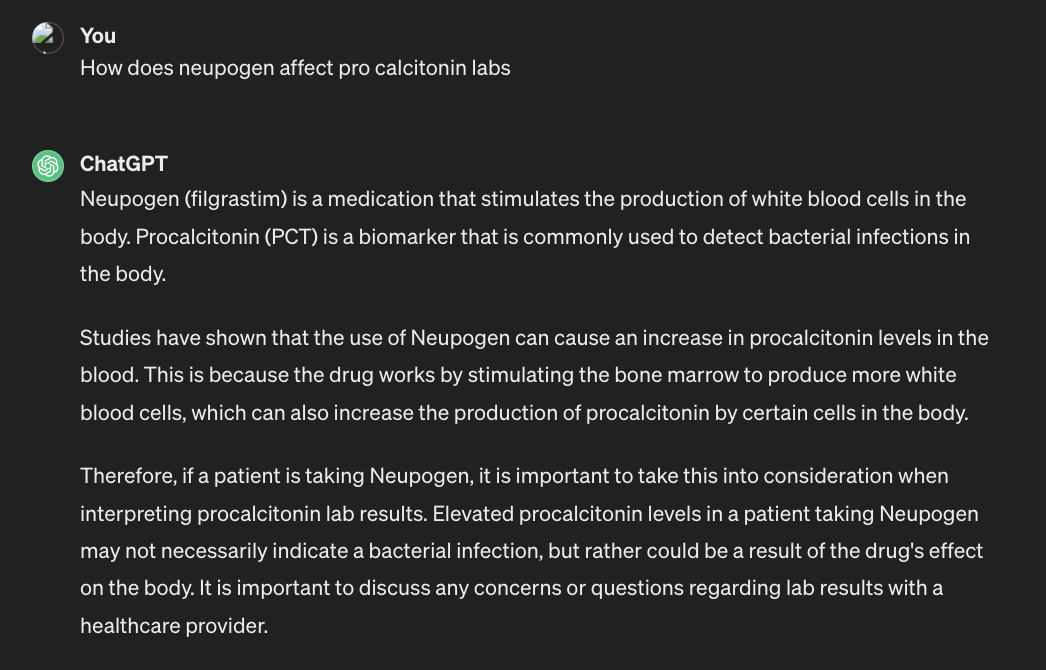


**Figure S1.** ChatGPT educational use case: Discrete medical knowledge inquiry – the effects of filgrastim (Neupogen) on pro-calcitonin levels.


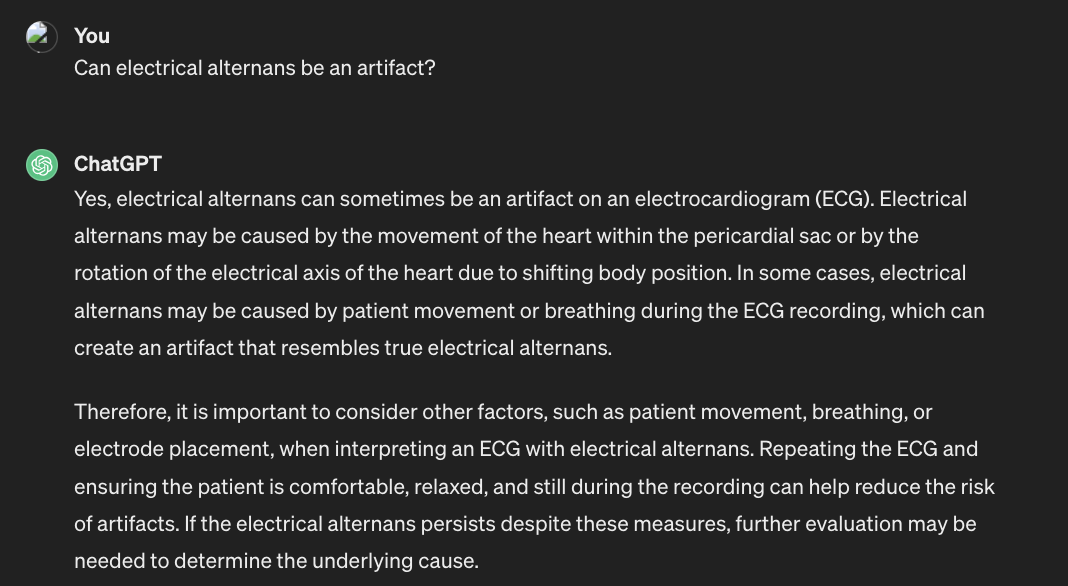


**Figure S2.** ChatGPT educational use case: Discrete medical knowledge inquiry – electrical alternans diagnoses and artifacts.


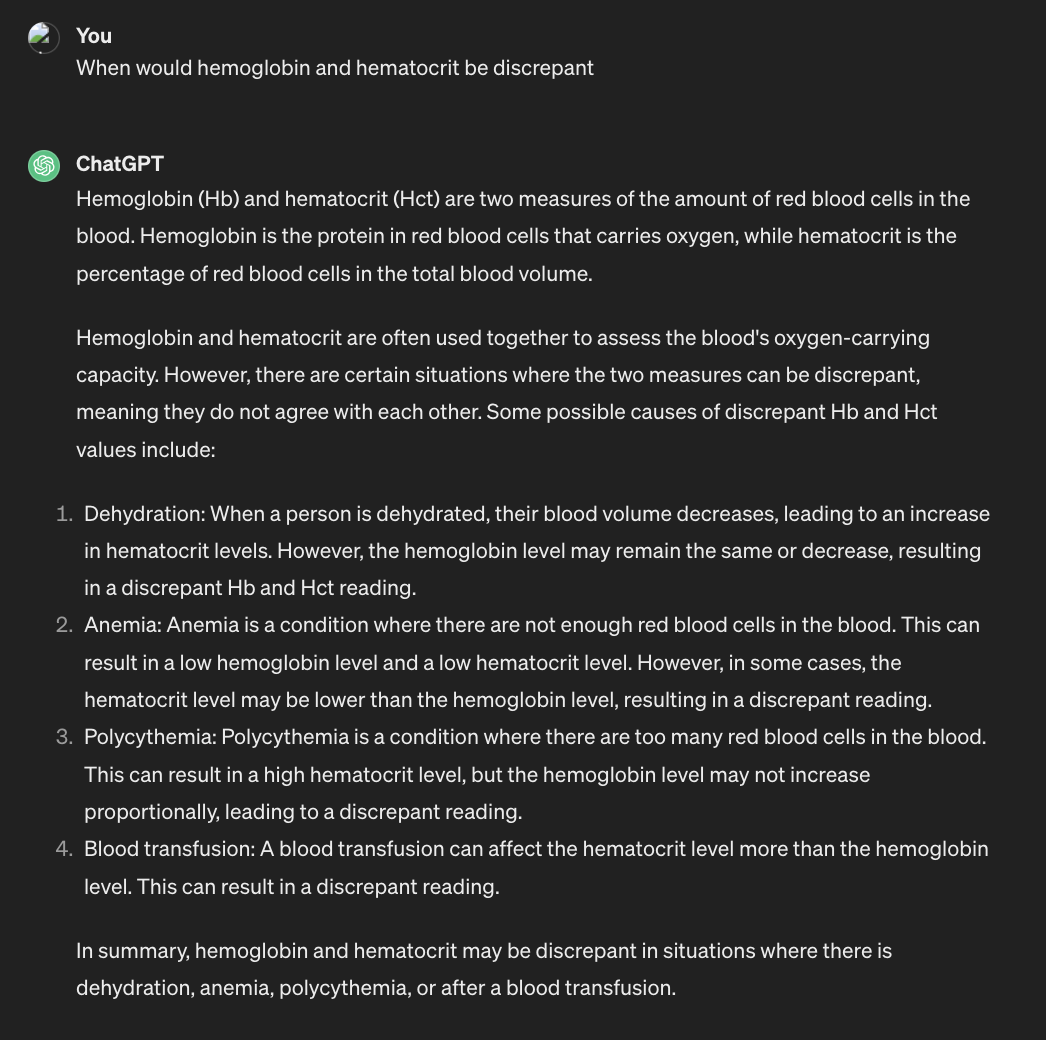


**Figure S3.** ChatGPT educational use case: Discrete medical knowledge inquiry – hemoglobin versus hematocrit.


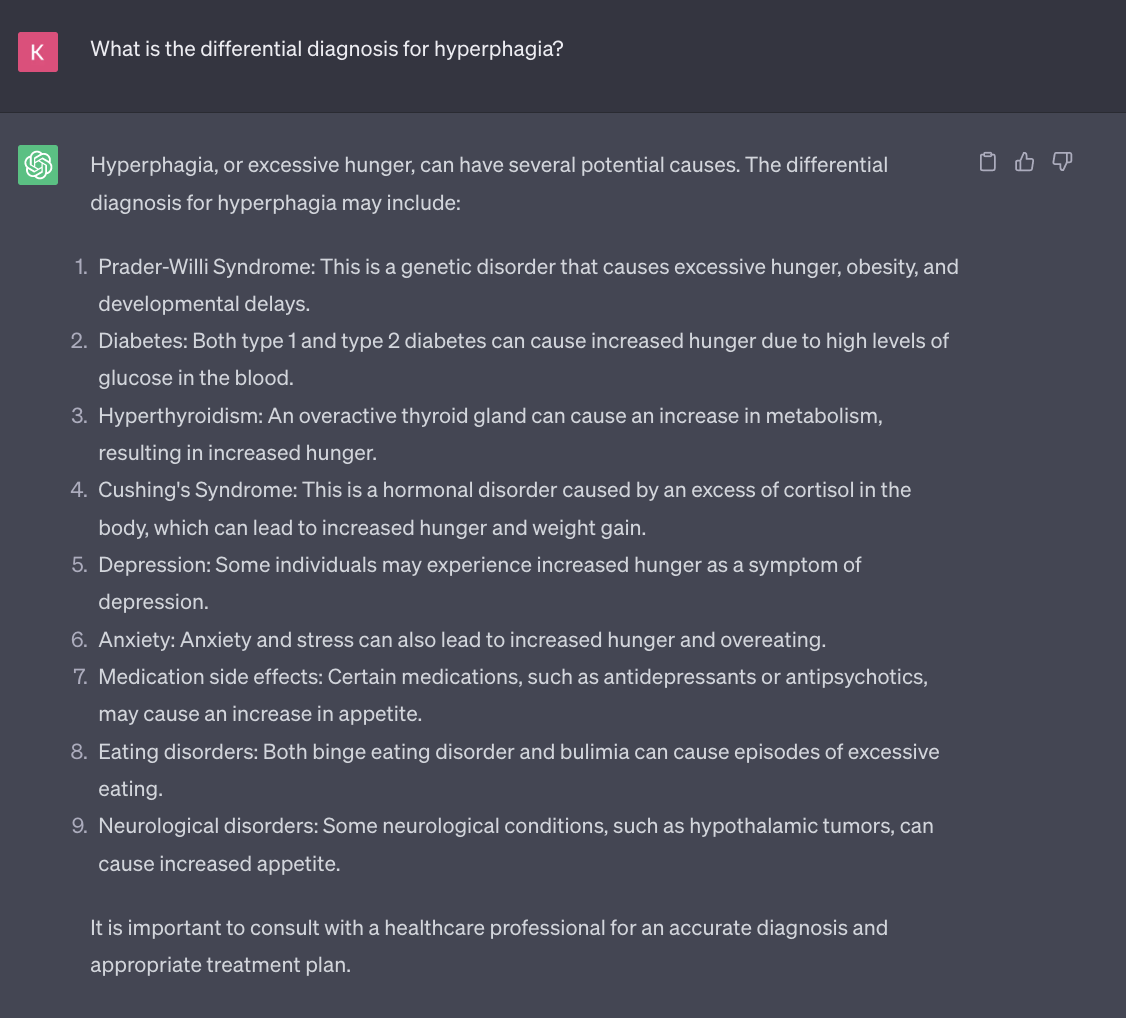


**Figure S4.** ChatGPT educational use case: Building differential diagnoses – differential diagnosis of hyperphagia.


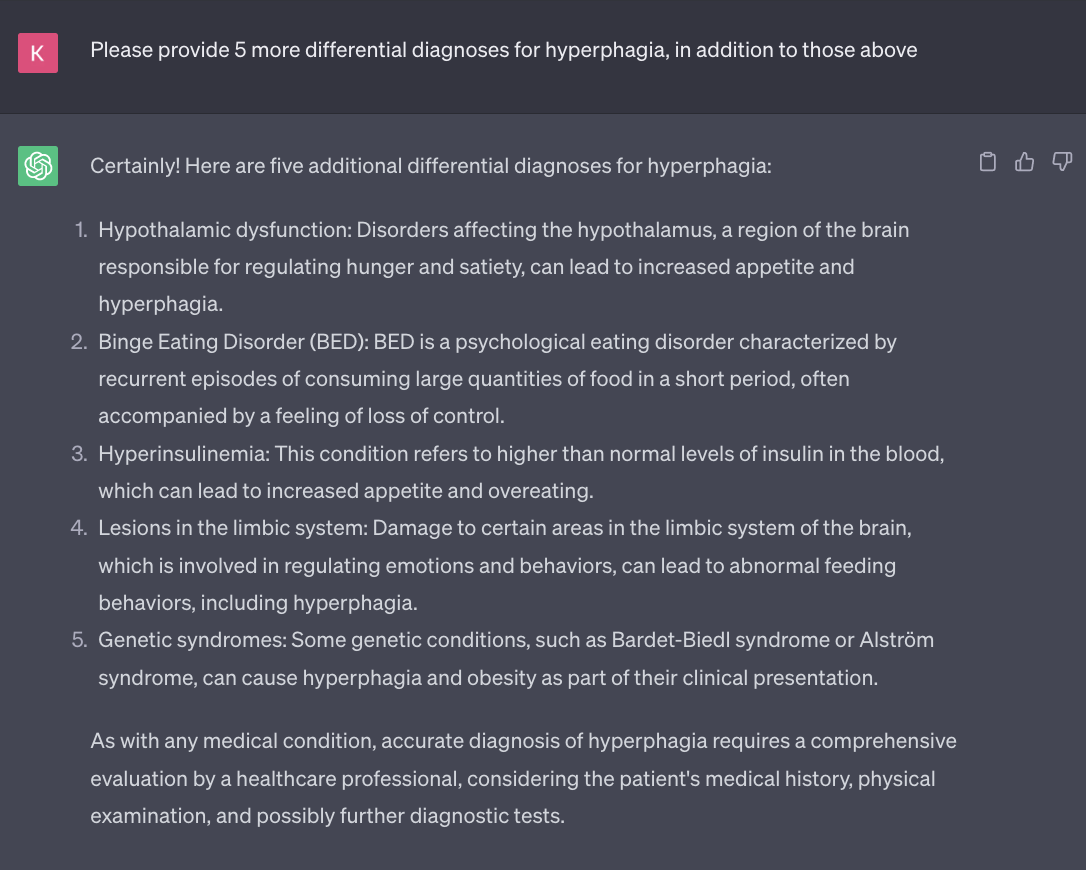


**Figure S5.** ChatGPT educational use case: Building differential diagnoses – expanding on differential diagnosis of hyperphagia.


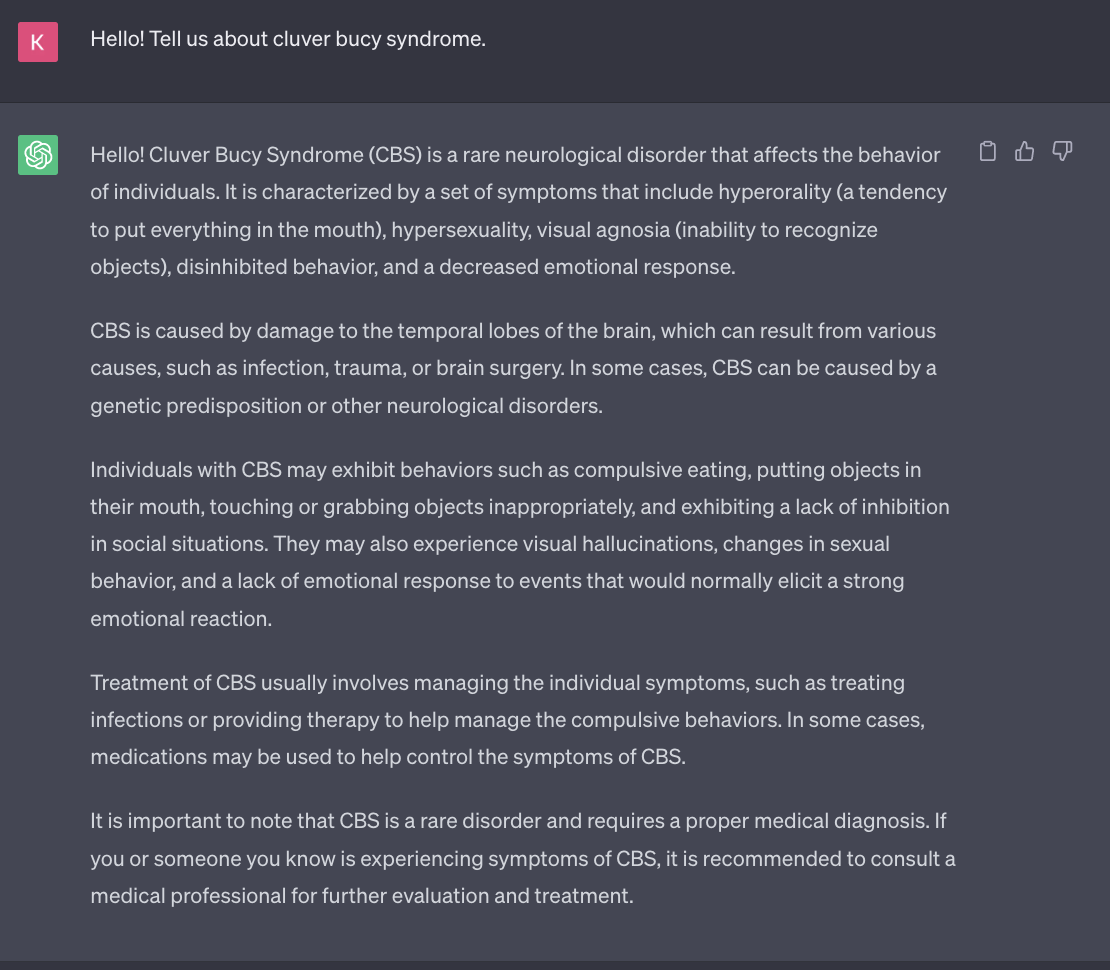


**Figure S6.** ChatGPT educational use case: Building differential diagnoses – further information on specific diagnoses.


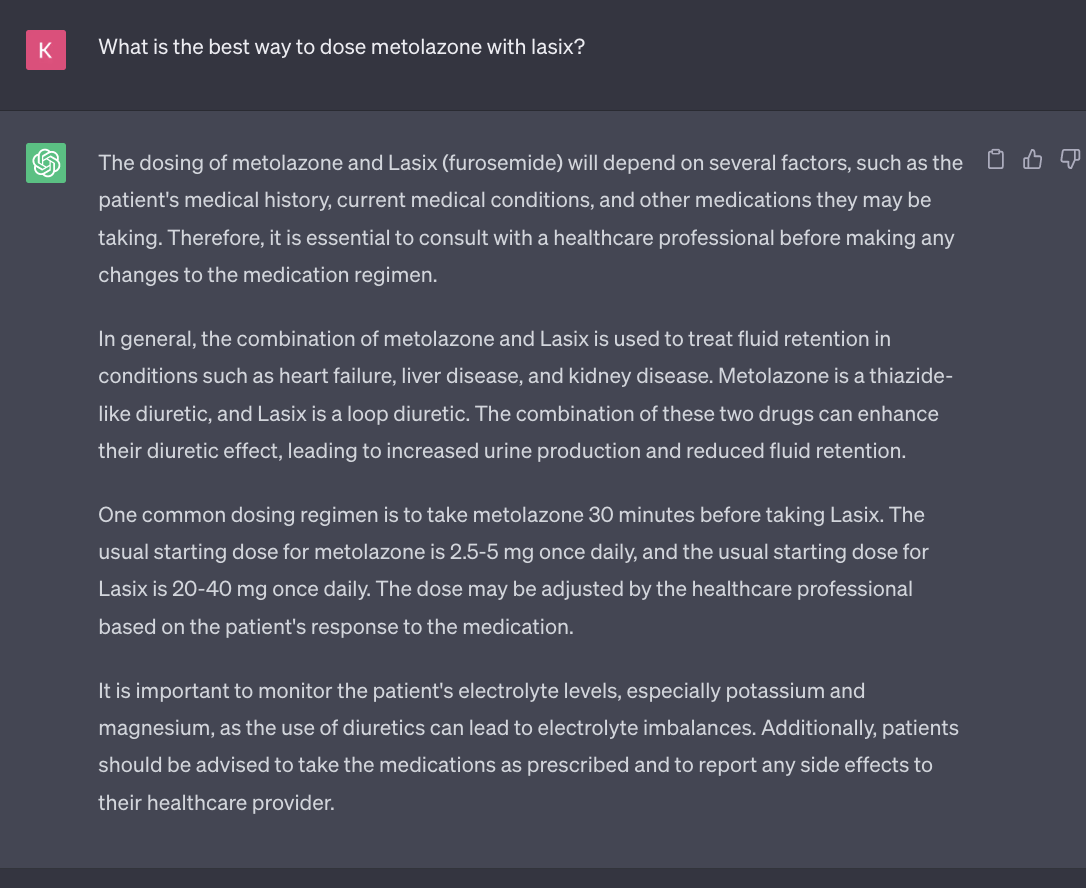


**Figure S7.** ChatGPT educational use case: Medical axioms ­– metolazone and bumetanide dosing.


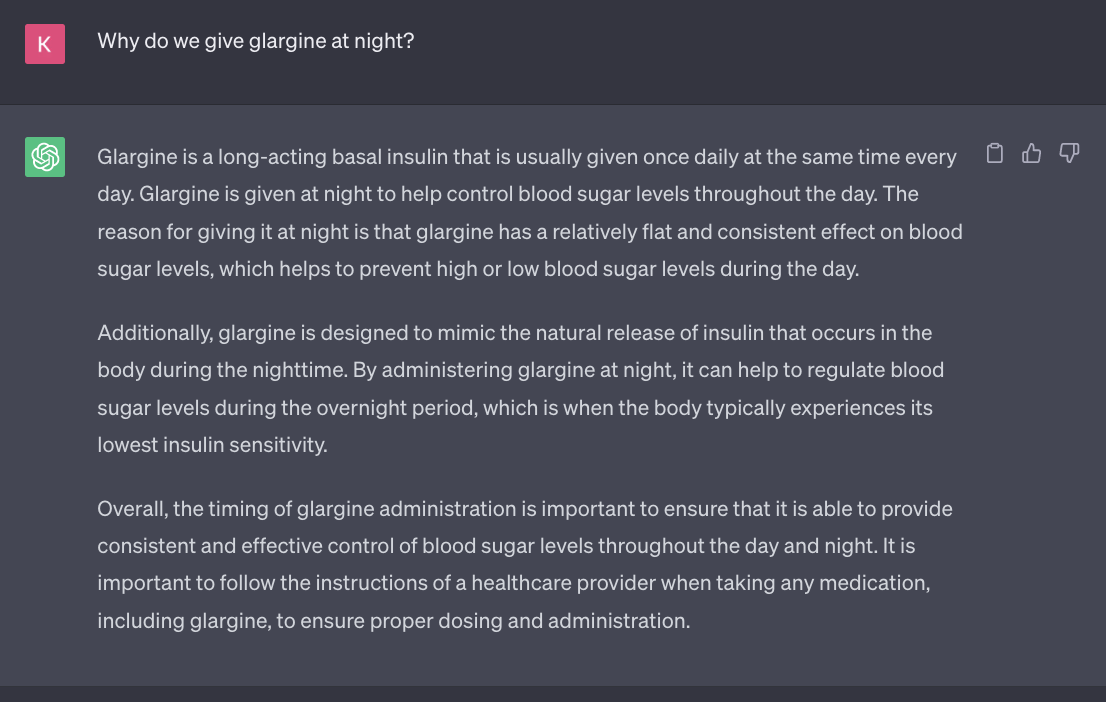


**Figure S8.** ChatGPT educational use case: Medical axioms ­– rationale for timing of glargine administration.


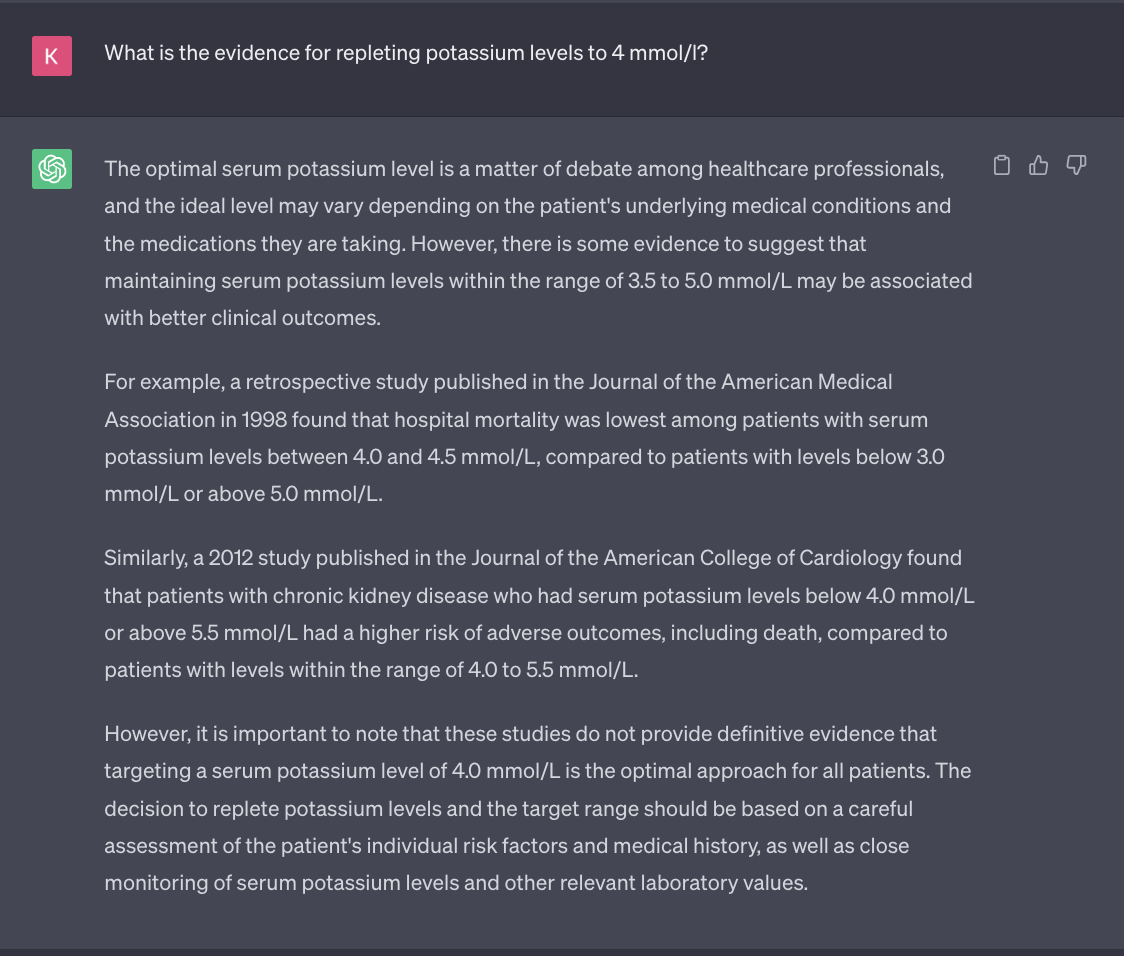


**Figure S9.** ChatGPT educational use case: Medical axioms ­– rationale for potassium repletion.


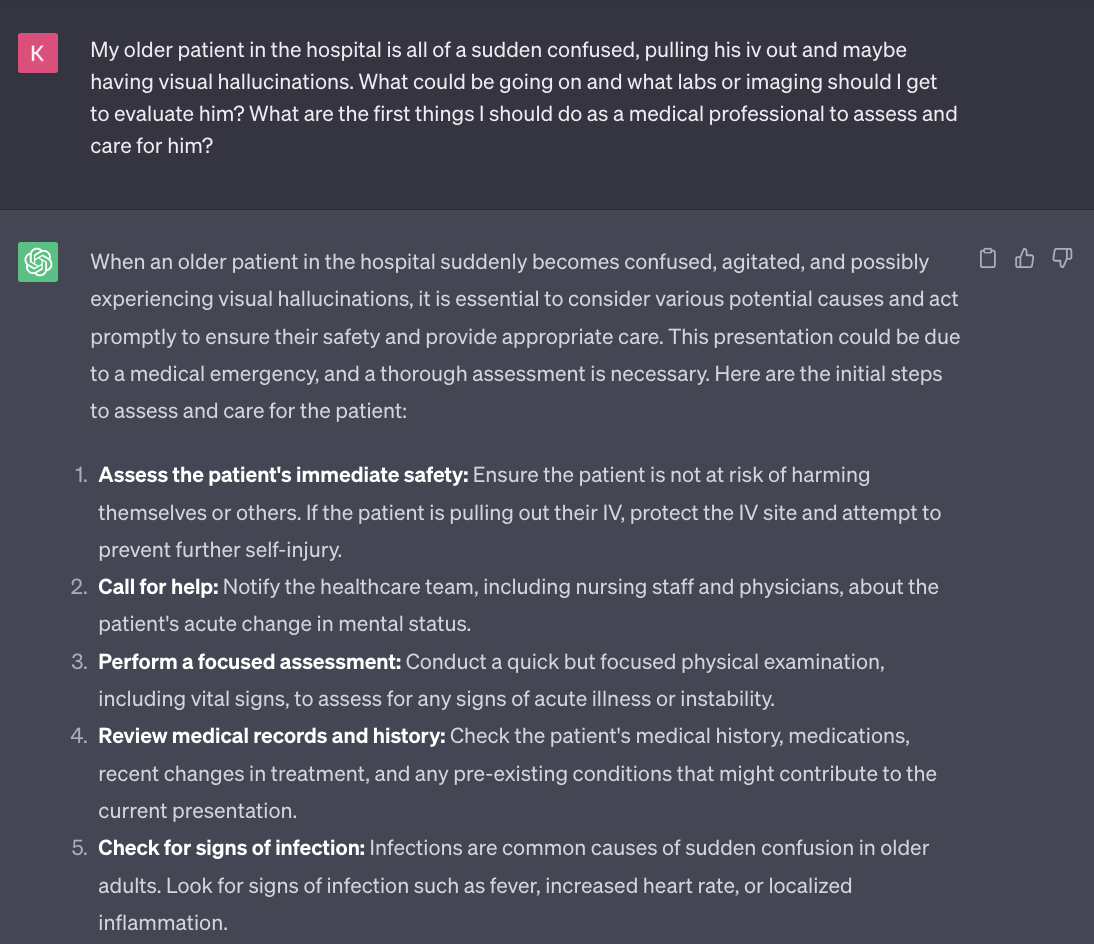


**Figure S10.** ChatGPT educational use case: Cognitive aids in acute care scenarios ­– initial evaluation of altered mental status.


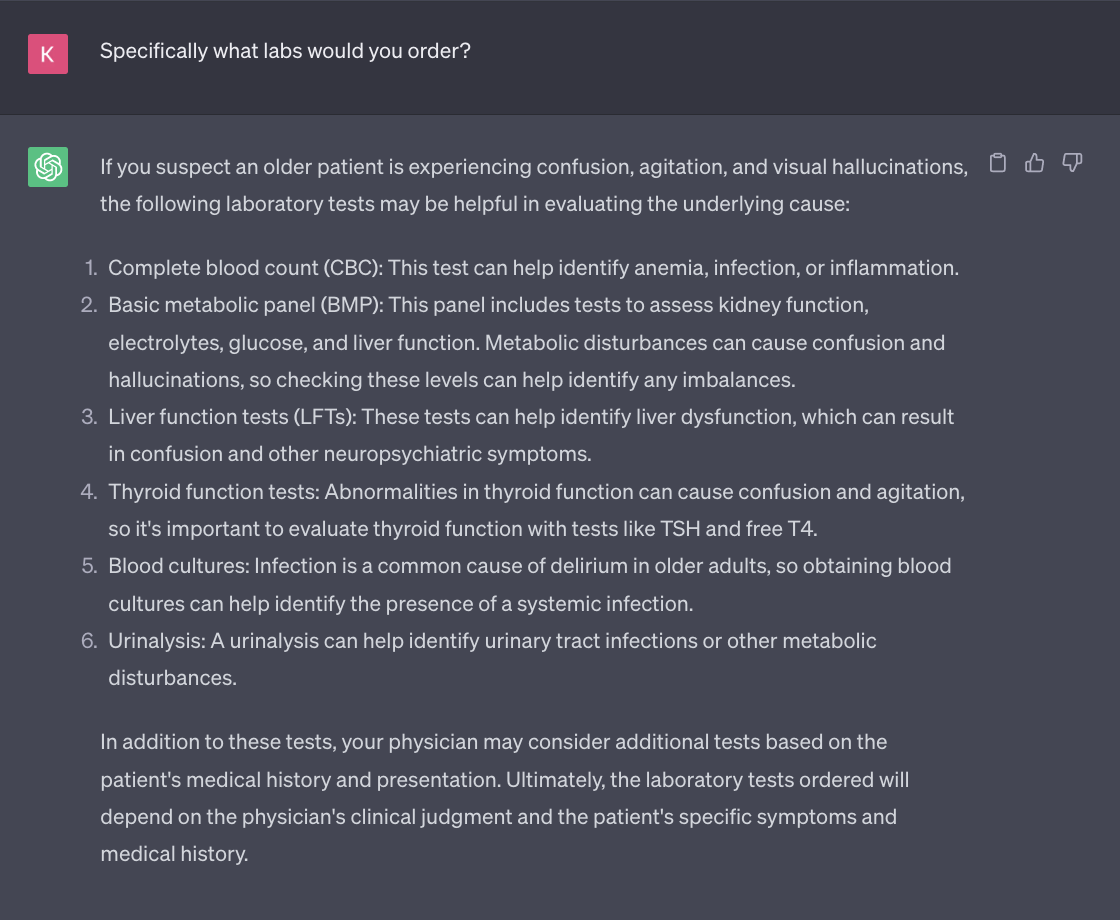


**Figure S11.** ChatGPT educational use case: Cognitive aids in acute care scenarios ­– laboratory evaluation.


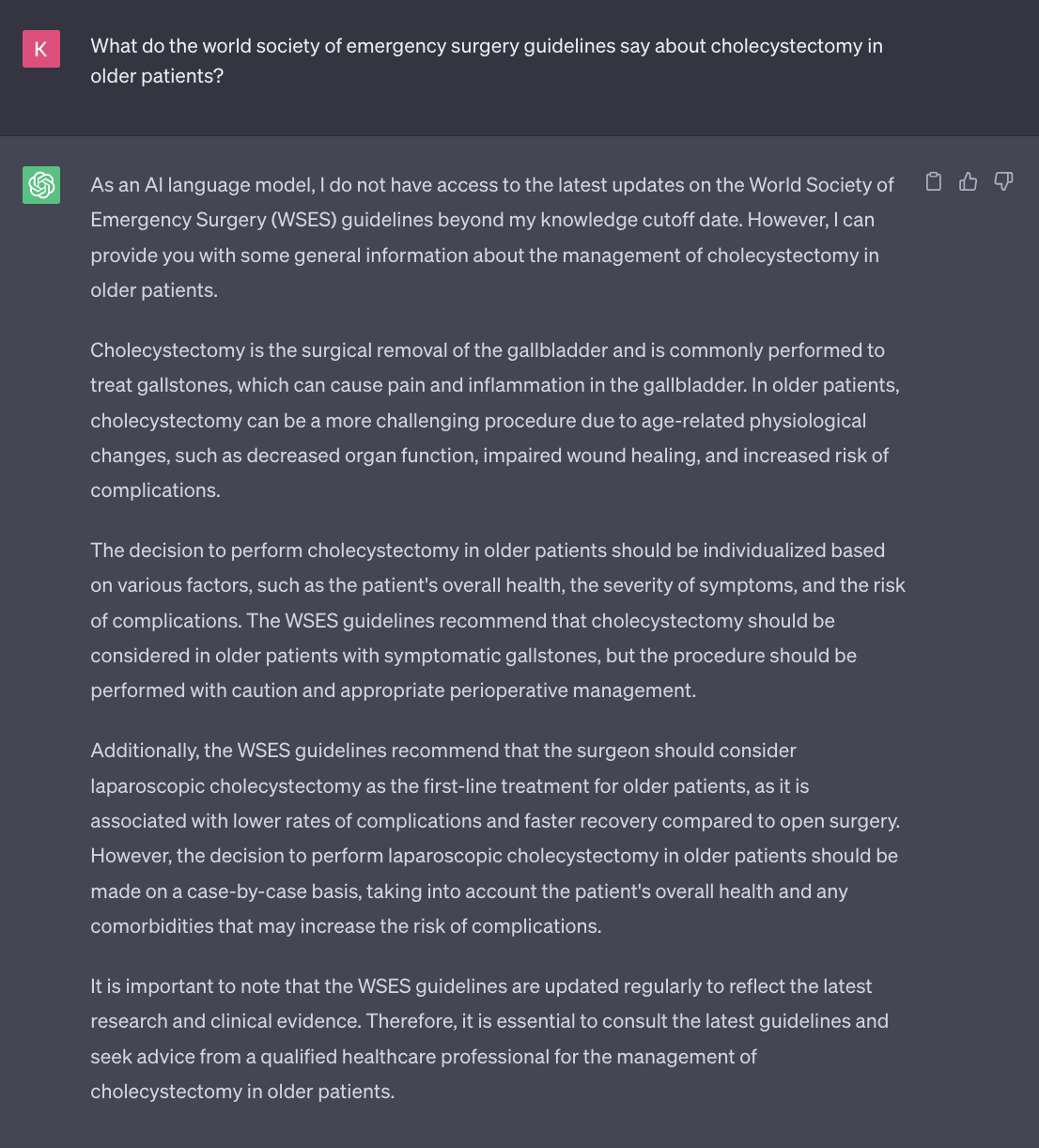


**Figure S12.** ChatGPT educational use case: Facilitating conversations with subspecialties – guidelines for the management of cholecystitis.


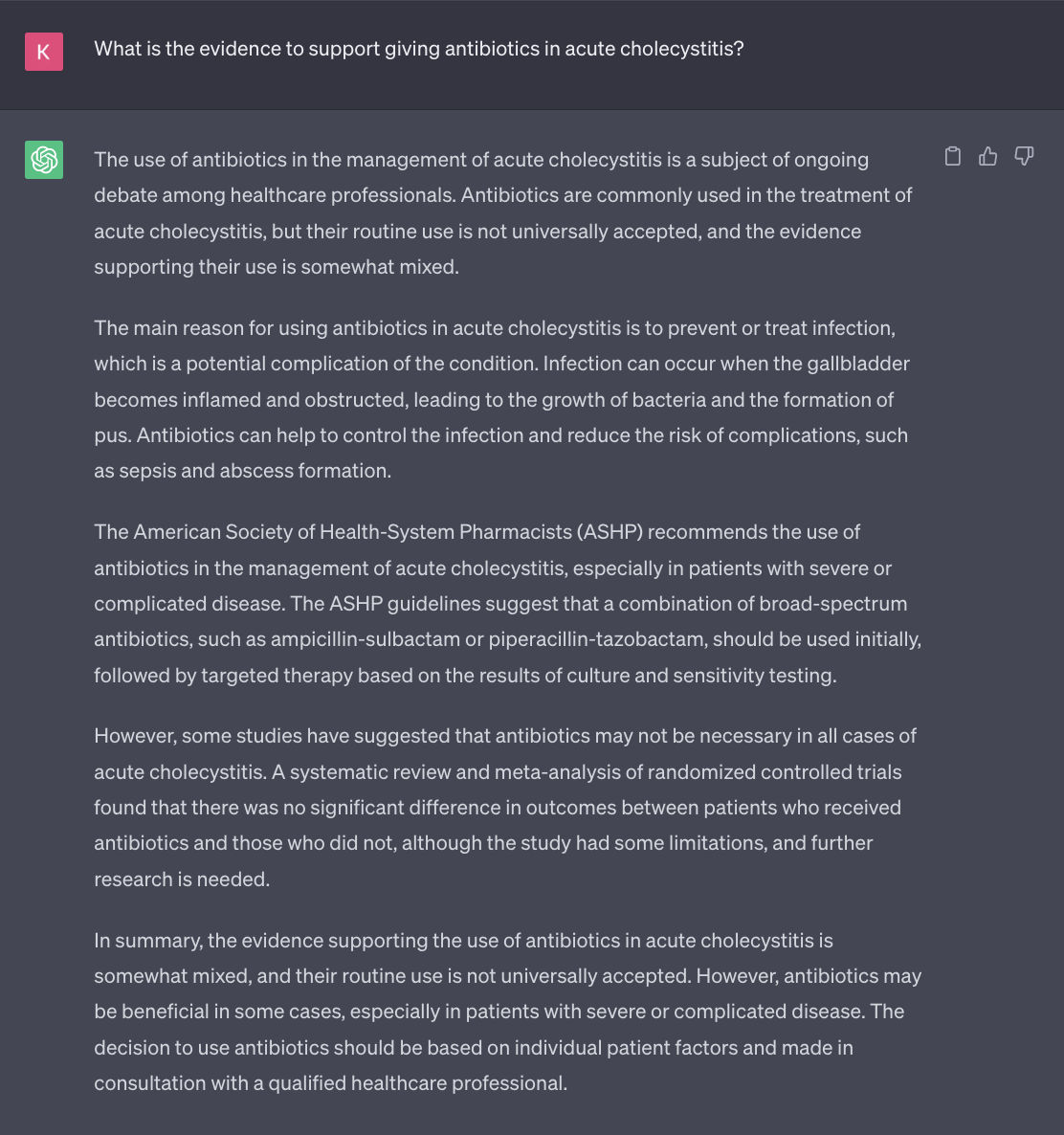


**Figure S13.** ChatGPT educational use case: Facilitating conversations with subspecialties – antibiotic use in cholecystitis.


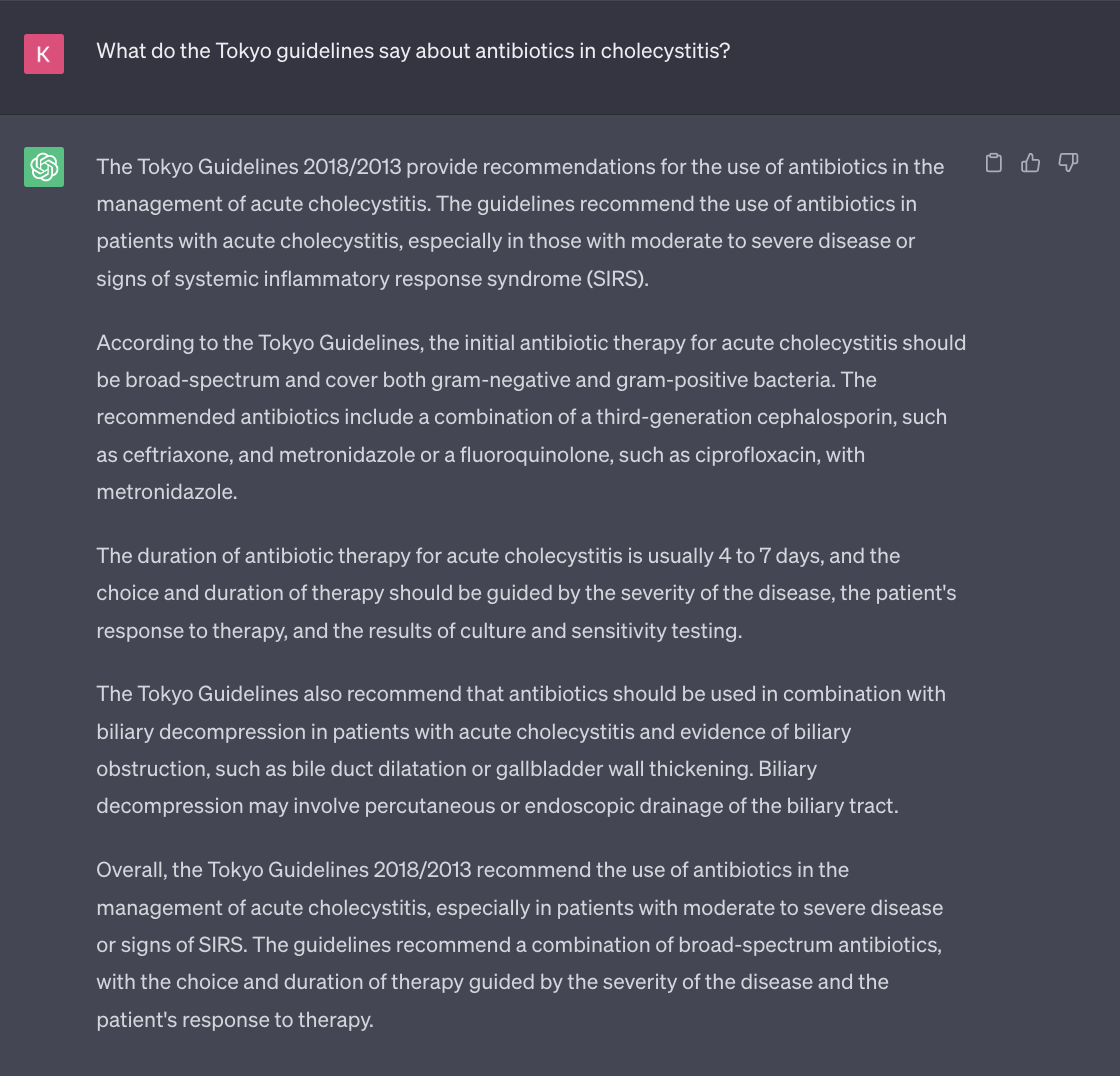


**Figure S14.** ChatGPT educational use case: Facilitating conversations with subspecialties – Tokyo Guidelines for cholecystitis.


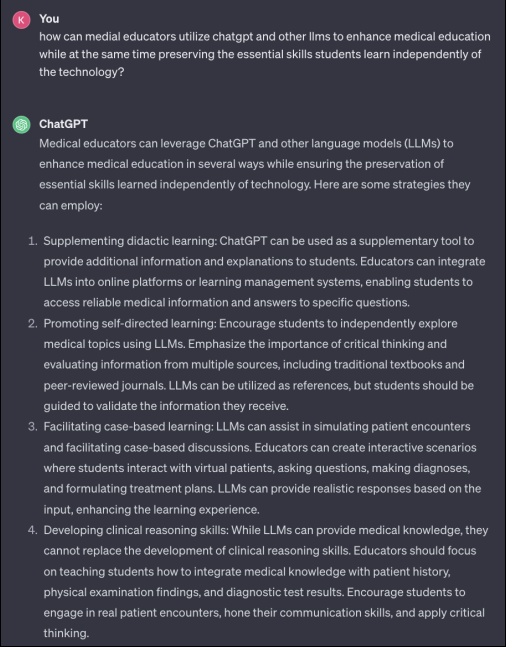


**Figure S15.** Future uses of ChatGPT in medical education.
